# A Systematic Hierarchical Virtual Screening Model for RhlR Inhibitors Based on PCA, Pharmacophore, Docking, and Molecular Dynamics

**DOI:** 10.3390/ijms25148000

**Published:** 2024-07-22

**Authors:** Jiarui Du, Jiahao Li, Juqi Wen, Jun Liu, Haichuan Xiao, Antian Zhang, Dongdong Yang, Pinghua Sun, Haibo Zhou, Jun Xu

**Affiliations:** 1 College of Pharmacy, Jinan University, Guangzhou 511436, China; djr2003@stu2021.jnu.edu.cn (J.D.); ljhhhh@stu2021.jnu.edu.cn (J.L.); wenjuqi97@stu.jnu.edu.cn (J.W.); liujun__jnu@163.com (J.L.); xhc991288@stu2021.jnu.edu.cn (H.X.); zat2021102909@stu2021.jnu.edu.cn (A.Z.); a970216469@stu2021.jnu.edu.cn (D.Y.); tsunph@jnu.edu.cn (P.S.); 2 State Key Laboratory of Bioactive Molecules and Druggability Assessment, Jinan University, Guangzhou 510632, China; 3 Key Laboratory of Xinjiang Phytomedicine Resource and Utilization, Ministry of Education, School of Pharmacy, Shihezi University, Shihezi 832003, China

**Keywords:** quorum sensing, RhlR, SAR, virtual screening, pharmacophore modeling, molecular docking, molecular dynamics

## Abstract

RhlR plays a key role in the quorum sensing of *Pseudomonas aeruginosa*. The current structure–activity relationship (SAR) studies of RhlR inhibitors mainly focus on elucidating the functional groups. Based on a systematic review of previous research on RhlR inhibitors, this study aims to establish a systematic, hierarchical screening model for RhlR inhibitors. We initially established a database and utilized principal component analysis (PCA) to categorize the inhibitors into two classes. Based on the training set, pharmacophore models were established to elucidate the structural characteristics of ligands. Subsequently, molecular docking, molecular dynamics simulations, and the calculation of binding free energy and strain energy were performed to validate the crucial interactions between ligands and receptors. Then, the screening criteria for RhlR inhibitors were established hierarchically based on ligand structure characteristics, ligand–receptor interaction, and receptor affinity. Test sets were finally employed to validate the hierarchical virtual screening model by comparing it with the current SAR studies of RhlR inhibitors. The hierarchical screening model was confirmed to possess higher accuracy and a true positive rate, which holds promise for subsequent screening and the discovery of active RhlR inhibitors.

## 1. Introduction

Quorum sensing (QS) is an essential regulatory system for biofilm formation and the expression of virulence factors in bacteria [1,2], and it also mediates antibiotic resistance [3,4]. *Pseudomonas aeruginosa* employs multiple types of signaling systems that constitute a QS network, regulating diverse biological functions, including las, rhl, and pqs [5]. LuxI/R plays a canonical role in the QS regulatory system, influencing various bacterial processes [6,7]. In this system, LuxI synthesizes acyl-homoserine lactone (AHL) signaling molecules, and LuxR interacts with AHL, binding to a specific DNA sequence known as the lux box. This interaction controls the expression of downstream genes related to virulence and physiological activities in bacteria [8]. Quorum sensing inhibitors (QSIs) can interfere with the QS process by blocking cognate AHL signals through structural similarities [9]. Currently, they are recognized as a promising strategy to treat bacterial infections and reduce antibiotic resistance [10,11].

In recent years, the Rhl system, as one of the LuxI/R systems, has garnered considerable attention in P. aeruginosa. Increasing evidence suggests that the Rhl system holds a dominant position in the regulatory hierarchy of QS. It has been discovered that Rhl mediates cellular toxicity in chronic infections independently [12,13,14]. Additionally, in environments that mimic infection conditions, the Rhl system assumes a pivotal role in QS regulation [15]. Comprising RhlR and RhlI enzymes, the Rhl system involves the former binding to the autoinducer N-butyryl-L-homoserine lactone (BHL) produced by the latter. Virulence factors reliant on QS, such as pyocyanin, lectin, rhamnolipids [16,17,18,19,20], are primarily activated by the binding complex of RhlR and its natural ligand BHL. This underscores the significance of the Rhl system in directing QS commands to regulate critical bacterial functions regarding infection and drug resistance [21,22]. Consequently, compounds capable of inhibiting binding to RhlR hold substantial importance. Several studies have reported RhlR inhibitors based on structural modifications [23,24,25,26,27], which primarily focus on elucidating the structure–activity relationship (SAR) at the ligand level. However, a SAR amalgamating ligand structure, ligand–receptor interactions, and receptor affinity are indispensable for uncovering potential RhlR inhibitors.

SAR can be explored through virtual screening techniques, which have found widespread application in the development of novel drugs [28,29,30,31]. Virtual screening encompasses various methods, such as pharmacophore modeling, molecular docking, and molecular dynamics. However, approaches reliant on a single tier often exhibit inherent limitations [32]. Collaborative virtual screening methods, capable of assessing active compounds from multiple dimensions, including ligand structural characterization, receptor–ligand interactions, and receptor affinity, offer enhanced potential and advantages in lead compound discovery. Leveraging SAR studies, our research group has contributed to the development of QSIs [33,34,35,36,37,38]. Recognizing the significance of SAR and virtual screening models for QSI development, our team aims to establish a multi-level screening system targeting core QS sub-systems. We have successfully devised a multi-level screening model targeting the pqs system [39].

In this study, we presented a systematic, hierarchical virtual screening model for inhibitors targeting RhlR. Initially, we established a database and utilized principal component analysis (PCA) to categorize available RhlR inhibitors. A systematic review was conducted accordingly. Based on the training sets, we employed pharmacophore modeling to elucidate the structural characteristics of active inhibitors. Subsequently, we examined the crucial interactions between ligands and receptors through molecular docking and molecular dynamics simulations, along with the calculation of binding free energy and strain energy. Moreover, to further contrast our study with existing research, relevant virtual screening criteria were established based on the described SAR. Test sets were then utilized to validate diverse screening criteria. The systematic hierarchical screening model established herein for RhlR inhibitors exhibits promise in identifying potential candidates.

## 2. Results

### 2.1. Establishment and Classification of the Database

#### 2.1.1. Principal Component Analysis (PCA) of the Database

We conducted a keyword search of existing literature and established criteria for inclusion and exclusion. Compliance with these criteria is summarized in Table S1.

**Figure 1:**
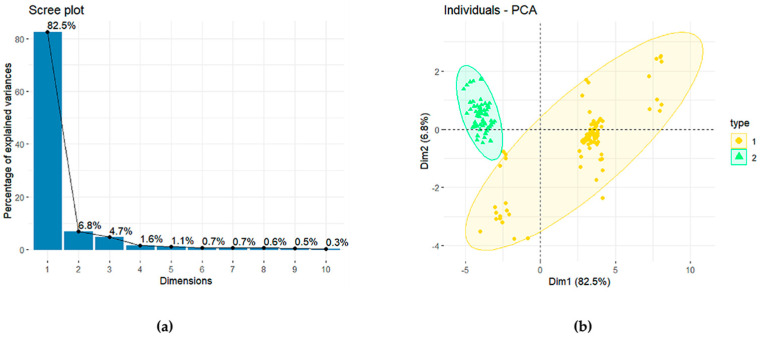
(**a**) Principal Components (PCs) contribution (**b**) Classification results of PCA (dim: dimension, PCA: principal component analysis).

All inhibitors identified in the eligible literature were included, thus establishing a database of RhlR inhibitors. To further explore the database, principal component analysis (PCA) was employed. We generated a series of molecular descriptors for the compounds and selected the top 20 ranked by Analysis of Variance (ANOVA) as the variables in PCA (Table S2). The correlation between the generated principal components (PCs) and the original variables is illustrated in Figure 1a. The results of the PCA, as shown in Figure 1b, demonstrate a distinction among the compounds. Consequently, all the RhlR inhibitors in the database were divided into two classes (Table S3), comprising 94 compounds for the first category and 68 for the second.

#### 2.1.2. Systematic Review

**Figure 2:**
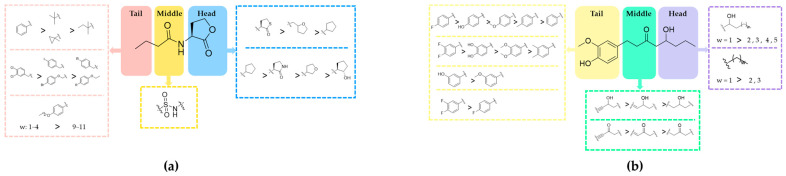
(**a**) Systematic review of Class 1; (**b**) Systematic review of Class 2.

The eligible literature was analyzed for the description of SAR and their evaluation methods, with the baseline table provided in Table S4. Subsequently, we delved into the SAR of RhlR inhibitors by categorizing compounds into three segments: head, middle, and tail. The SAR result for Class 1 is as follows (Figure 2a). In the head region, homoserine lactone, homocysteine thiolactone, tetrahydrofurfuryl ring, cyclopentane, furan ring, cyclolactam, and benzene ring all exhibit potent antagonistic activity [40]. Tetrahydrofurfuryl rings and cyclopentanes demonstrate stronger substitution activity compared to cyclolactam [23]. The central region is mainly characterized by amide groups commonly found in BHL. Modification of sulphonamide can increase inhibitory activity by increasing the spatial volume of amide [23]. In the tail region, various antagonistic group modifications can be utilized, including hydrocarbons, aryl rings, and cyclohexyl groups containing quaternary or sp2 hybridized α-carbons, all of which possess inhibitory activity. Aryl substituents in the tail primarily consist of phenylmethyl, phenylethyl, and phenoxymethyl, sharing the common feature of substitutions, typically in the para-position of the aryl group (-I, -Br, -NO_2_, -CH_3_, -CF_3_, and -SCH_3_). Among these, halogenated substitutions exhibit higher antagonistic activity, with dichloro-substitutions at the 3,4-position of phenylmethyl demonstrating the strongest antagonistic effect [25]. Regarding Class 2 (Figure 2b), the head is often an alkyl chain, and its length is related to inhibition. Regardless of the presence of a β-hydroxy group, shorter alkyl chains exhibit higher inhibitory activity. And β-hydroxy substitution does not seem to be essential for the activity. The central region can be either a carbonyl or hydroxyl group, both of which are crucial for binding to RhlR. Variances in the bonds between the central and tail phenyl rings can be observed, with double bonds demonstrating stronger inhibitory activity compared to single bonds and alkynyl groups exhibiting higher binding affinity relative to their corresponding single or double bonds. The tail of compounds in Class 2 consistently features a benzene ring, with polar substitutions being crucial for inhibition. At the 3′-position, larger substituents exhibit weaker antagonistic effects. Small polar groups at the 4′-position are significant, as exemplified by the -OH group in the parent nucleus of gingerol. In most cases, -F has been demonstrated to be an optimal substituent.

#### 2.1.3. Establishment of Training Sets and Test Sets

Training and test sets were then constructed separately for the two classes. For each class of inhibitors, the top 10 compounds demonstrating inhibitory activity were designated as active, while the bottom 10 were classified as inactive, forming the training sets for pharmacophore analysis, molecular docking, and molecular dynamics simulations (Table S5). Subsequently, for the test set, we selected the top 30 compounds from the first class and the top 20 from the second as active compounds, excluding those already present in the training set. A set of 1000 decoys generated from www.dude.docking.org was designated as inactive compounds of the test set, with structural resemblance to active inhibitors (Table S6).

### 2.2. Building Models Based on Training Sets

#### 2.2.1. Pharmacophore Modeling

The pharmacophores were modeled using the training set. From the composite ranking, we selected AAADR_1 as the primary candidate pharmacophore for the first class of inhibitors (Table S7a). This pharmacophore comprises three hydrogen bond donors, a hydrogen bond acceptor, and an aromatic ring. Notably, these features align with the fundamental structure of BHL: the hydrogen bond donor corresponds to the oxygen atom (O) of the head lactone ring and middle amide, the hydrogen bond acceptor corresponds to the nitrogen atom (N) of the middle amide, and the aromatic ring corresponds to the tail benzene ring.

As for Class 2, AHHR_2 was selected as the optimal pharmacophore model (Table S7b), consisting of four chemical features: an aromatic ring, two hydrophobic features, and a hydrogen bond acceptor. Likewise, gingerol, the parent nucleus of Class 2, matches these elements, with a hydrophobic carbon chain fitting H in the head, a carbonyl fitting A in the middle, and a para-substituted benzene ring fitting R and H in the tail.

**Figure 3:**
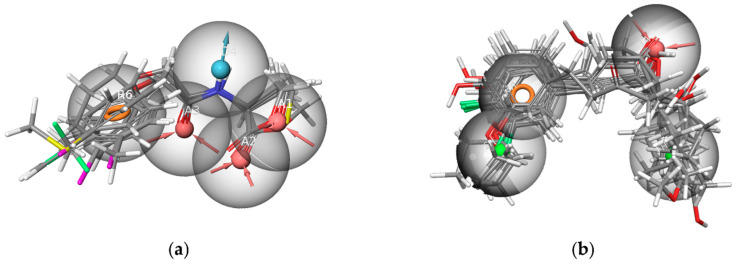
(**a**) The match between the pharmacophore AAADR_1 and active compounds in Class 1; (**b**) The match between the pharmacophore AHHR_2 and active compounds in Class 2 (the red sphere represents the hydrogen bond acceptor, the blue sphere represents the hydrogen bond donor, the green sphere represents the hydrophobic feature, and the orange circle represents the aromatic ring).

The spatial arrangements of the pharmacophores are depicted in Figure 3. All active compounds in each training set perfectly matched their respective pharmacophores, while inactive compounds exhibited only partial alignment, suggesting the efficacy of both pharmacophore models in recognizing the structural characteristics.

#### 2.2.2. Molecular Docking, Molecular Dynamics Simulations, Calculations of Binding Free Energy and Strain Energy

**Figure 4:**
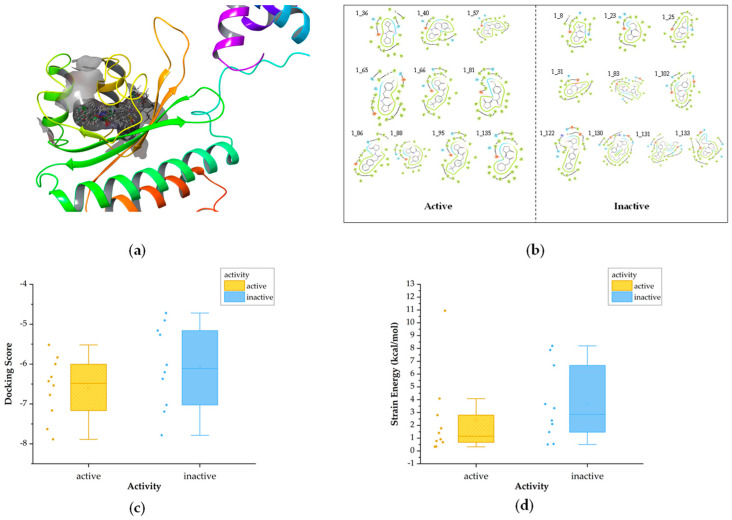
Diagram of molecular docking of Class 1. (**a**)The 3D docking diagram of compounds in protein pocket. (**b**) Diagram of key interaction sites for docking. (**c**)The box plot of docking scores. (**d**) The box plot of strain energy (in Figure 4c,d, the yellow bars represent active compounds, while the blue bars represent the inactive).

Molecular docking of compounds from the training set into the RhlR protein (PDB ID: 8B4A) was conducted in order to preliminary determine the interactions of receptor-active compounds and their binding sites (Figure 4a).

For Class 1, we initially analyzed the receptor–ligand interactions of the molecules in the training set during docking and observed that the amide bond positioned at the center played a crucial role in forming hydrogen bonding with amino acid residues (Figure 4b). Figure 4c presents box plots comparing the docking scores between active and inactive compounds. The docking scores of active compounds are slightly lower than those of inactive compounds, indicating a superior binding affinity. And it can be seen that all active compounds demonstrated docking scores below −5.5. Strain energy is further calculated to explore conformational changes in compounds, with lower values indicating less energy consumption in binding to the receptor. The results indicate that active inhibitors exhibit a greater propensity for binding (Figure 4d).

**Figure 5:**
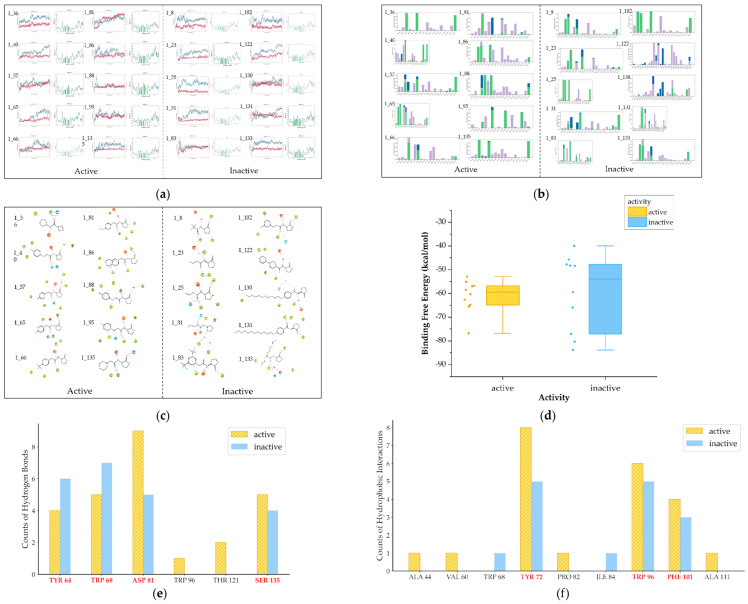
Diagram of molecular dynamics of Class 1 (**a**) RMSD and RMSF plots (**b**) Interaction histograms of compounds (**c**) The contact sites of interactions for compounds (**d**) The box plot of binding free energy (**e**) The bar chart of key hydrogen bonds (**f**) The bar chart of key hydrophobic interactions (in Figure 5d–f, the yellow bars represent active compounds, while the blue bars represent the inactive).

To enhance our insight into interactions between ligands and receptors, we conducted molecular dynamics simulations on the training set of Class 1. This approach facilitates a more precise determination of the crucial RhlR receptor–ligand interactions through advanced calculations and allows for an examination of interaction stability over time. The binding states of the compounds were simulated within a 100 ns timeframe of each other. RMSD plots exhibiting fluctuations within 3 Å are considered indicative of good stability. It is observed that all compounds remained relatively stable for 50–100 ns (Figure 5a), affirming the validity of the selected dominant conformation. Interactions with strengths exceeding 0.4 are identified as strong interactions (Figure 5b). Most active compounds in Class 1 demonstrated a propensity to form stable, strong hydrogen-bonding interactions with the amino acids TYR 64, TRP 68, ASP 81, and SER 135, as shown in Figure 5e. The observation that these interactions predominantly occur via the amide bond further corroborates the findings from the docking study, underscoring the indispensability of the amide bond for receptor binding in Class 1. Specifically, the N in the amide bond often forms bonds with ASP_81, whereas the O frequently interacts with TYR 64, TRP 68, and SER 135. And the O of lactones is also a crucial site for hydrogen bond formation (Figure 5c). Moreover, the active compounds exhibited prolonged hydrophobic interactions with TYR 72, TRP 96, and PHE 101 (Figure 5f). These interactions are likely facilitated by the presence of hydrophobic benzene rings or elongated chains in compounds.

We also performed binding free energy calculations (Figure 5d). The binding free energy of active compounds is consistently below −50 kcal/mol, whereas that of inactive compounds exhibits a broader range, typically indicating thermal instability.

Therefore, regarding the first class of RhlR inhibitors, the screening criteria can be summarized as follows:

1. Compounds should form hydrogen bonds with at least two of the following amino acid residues: TYR 64, TRP 68, ASP 81, and SER 135;
2. Compounds should engage in hydrophobic interactions with TYR 72, TRP 96, and PHE 101;
3. The binding free energy should ideally be less than −50 kcal/mol;
4. Docking scores should be below −5.5.

**Figure 6:**
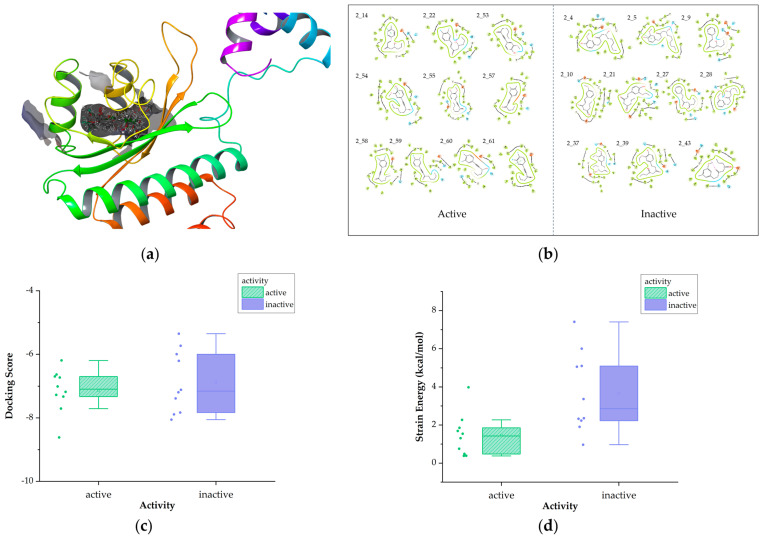
Diagram of molecular docking of Class 2 (**a**) The 3D docking diagram of compounds in protein pocket (**b**) Diagram of key interaction sites for docking (**c**) The box plot of docking scores (**d**) The box plot of strain energy (in Figure 6c,d, the green bars represent active compounds, while the purple bars represent the inactive).

In Class 2, it can be observed that the head and tail regions of the compounds are surrounded by hydrophobic amino acids (Figure 6b), as this class of compounds typically features long chains or aromatic rings at either end. The result of docking scores is shown in Figure 6c. In this category, the docking scores of active molecules are generally less than −6 and lower than those of inactive ones, suggesting a higher binding affinity to the receptor. Strain energy was also calculated (Figure 6d). It is evident that the strain energy of active compounds is significantly lower, indicating less conformational change is required in binding.

**Figure 7:**
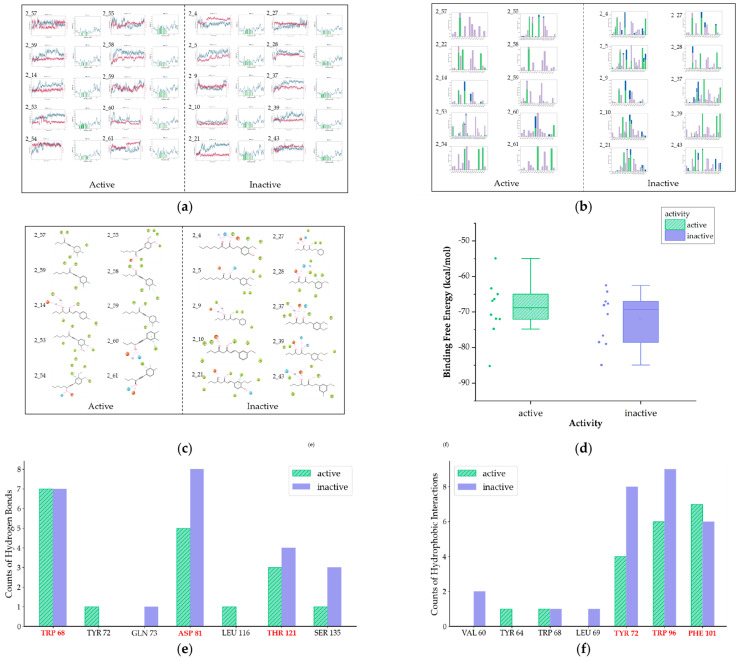
Diagram of molecular dynamics of Class 2 (**a**) RMSD and RMSF plots (**b**) Interaction histograms of compounds (**c**) The contact sites of interactions for compounds (**d**) The box plot of binding free energy (**e**) The bar chart of key hydrogen bonds (**f**) The bar chart of key hydrophobic interactions (in Figure 7d–f, the green bars represent active compounds, while the purple bars represent the inactive).

We further simulated the dynamic interactions of Class 2 (Figure 7a–c). The results indicate that while key interactions between active and inactive compounds are not significantly different, active compounds generally exhibit hydrogen bonding with TRP 68, ASP 81, and THR 121, as well as hydrophobic interactions with TYR 72, TRP 96, and PHE 101 (Figure 7e,f). The results of binding free energy are depicted in Figure 7d, with active compounds generally below −50 kcal/mol.

Hence, concerning the second class, the screening criteria can be encapsulated as given below:

1. Docking scores should ideally be below −6;
2. Strain energy should be less than 4 kcal/mol;
3. Compounds should form hydrogen bonds with TRP 68, ASP 81, or THR 121;
4. Compounds should engage in hydrophobic interactions with TYR 72, TRP 96, and PHE 101;
5. The binding free energy should be less than −50 kcal/mol.

### 2.3. Establishment of Virtual Screening Models for Existing SAR Study

**Figure 8:**
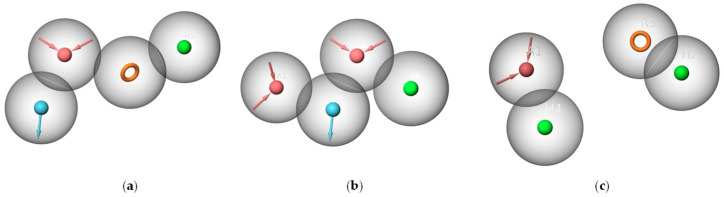
(**a**) Pharmacophore ADRH established based on the existing SAR (**b**) Pharmacophore AADH established based on the existing SAR (**c**) Pharmacophore AHHR established based on the existing SAR (the red sphere represents the hydrogen bond acceptor, the blue sphere represents the hydrogen bond donor, the green sphere represents the hydrophobic feature, and the orange circle represents the aromatic ring).

Several articles currently describe and summarize the structure–activity relationships (SAR) of RhlR inhibitors. For Class 1, intensive studies were concentrated on structural modifications of the parent nucleus at the ligand level. However, the specific pharmacophore has not been modeled. Therefore, we constructed it based on the key structures described in the literature. Eibergen et al. discovered that compounds exhibiting antagonistic activity typically feature para-substituted aromatic acyl groups [25]. Building upon this description, we developed an ADRH pharmacophore model (Figure 8a). In this model, the oxygen and nitrogen atoms of the amide in the central region serve as the hydrogen bond acceptor (A) and hydrogen bond donor (D), respectively, while the tail consists of a benzene ring (R) with a hydrophobic group (H) attached in the para position. Additionally, Boursier et al. proposed predominant structures for the head, middle, and tail segments following relevant modifications [23]. The pharmacophore AADH was also established accordingly, which perfectly matched three compounds characterized by dominant structures: compound 14 featuring a tetrahydrofurfuryl amine moiety in the head, compound 8 containing a sulphonamide moiety in the middle, and compound 5 exhibiting a tail closely matching the volume of the HL space (Figure 8b).

For Class 2, research has reported the SAR of inhibitors and further investigated the interactions between inhibitors and receptors. SangJin Nam et al. highlighted the significance of benzene rings with polar groups, as well as carbonyl or hydroxyl groups at the γ-position from the phenyl group, in binding with RhlR [27]. The reported inhibitors typically featured alkyl chains at the end. Based on the aforementioned SAR studies, we have similarly established the pharmacophore model AHHR (Figure 8c). Herein, R corresponds to a benzene ring substituted with a polar group, A corresponds to a carbonyl group, and H corresponds to an alkyl chain. The research also suggests that inhibitors primarily interact with TYR 72 through π-π stacking and form hydrogen bonds with TRP 68 when binding to RhlR. We designate this as the criterion in the ligand–receptor interaction aspect of this screening model.

### 2.4. Validating Models Based on Test Sets

#### 2.4.1. Pharmacophore Validation

**Figure 9:**
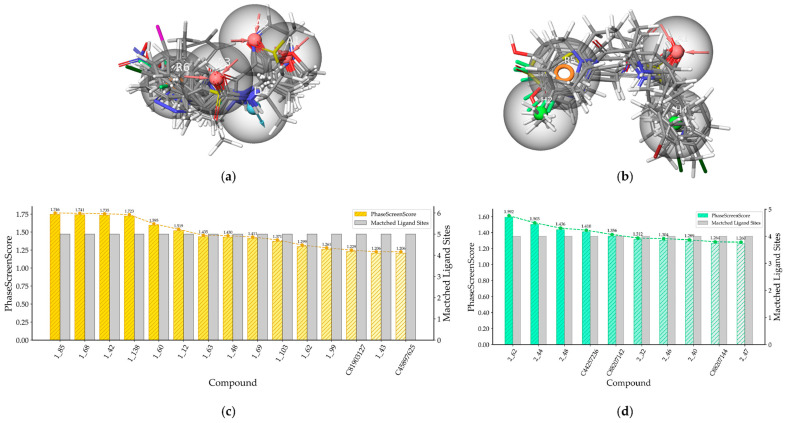
(**a**) The match between test set of Class 1 and the pharmacophore AAADR_1 (**b**) The match between test set of Class 2 and the pharmacophore AHHR_2 (**c**) The top-ranked compounds in test sets of Class 1 based on pharmacophore screening (the yellow bars represent the pharmacophore scoring of different compounds, while the gray bars represent the number of matches with pharmacophore elements) (**d**) The top-ranked compounds in test sets of Class 2 based on pharmacophore screening (the green bars represent the pharmacophore scoring of different compounds, while the gray bars represent the number of matches with pharmacophore elements).

The test sets of two types were, respectively, imported into the constructed pharmacophores AAADR_1 and AHHR_2 for the initial step of structure-based screening. The screening results of the pharmacophore are shown in Figure 9a,b. Among them, the top 15 compounds from Class 1 were selected as potential inhibitors, including 13 active and 2 inactive compounds (Figure 9c). For Class 2, the top 10 compounds were selected, comprising 7 active and 3 inactive compounds (Figure 9d). The retained compounds exhibited a perfect match with the characteristic elements of the pharmacophores, thus being considered as the test sets for the subsequent validation screening.

#### 2.4.2. Validation through Molecular Docking, Molecular Dynamics Simulations, and Binding Free Energy Calculations

**Figure 10:**
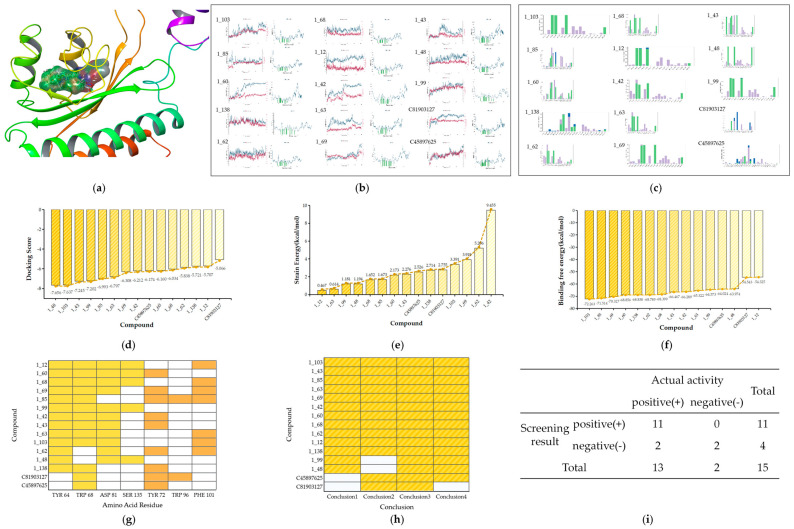
(**a**) The 3D docking diagram of test set of Class 1 (**b**) RMSD and RMSF plots of test set (**c**) Interaction histograms of test set (**d**) The ranking of compounds in Class 1 based on docking scores (**e**) Strain energy of test set of Class 1 (**f**) Binding free energy of test set of Class 1 (**g**) The matches of interactions with the standard of Class 1 (the blocks with yellow represent hydrogen bonding, while orange signify hydrophobic interactions) (**h**) The summary of screening results for test set of Class 1 (the blocks with yellow represents compliance with standards, while the absence of a color indicates non-compliance) (**i**) The screening performance of the model was validated by the test set of Class 1.

We further evaluated these compounds based on their receptor–ligand interactions. The screening criteria for Class 1 were initially validated. Figure 10a illustrates a 3D action diagram of the molecular docking for the test sets, while Figure 10d,e present the docking scores and strain energy, indicating that only C81903127 failed to meet the criteria established by the training set.

Subsequently, we conducted screening via molecular dynamics. Figure 10b depicts the RMSD and RMSF plots of the test set in Class 1, which remained relatively stable from 50 ns to 100 ns. Interaction histograms between the test set and the amino acid residues are presented in Figure 10c. Among them, 10 compounds met the key interaction conditions outlined in Section 2.2.2 simultaneously. However, compounds 1_48 and 1_99 lacked crucial hydrophobic interactions, and compounds C45897625 and C81903127 did not exhibit key hydrogen bonding interactions (Figure 10g). The results of binding free energy demonstrated that the compounds selected through key interaction screening also met the requirement for affinity (Figure 10f).

Summarizing the screening results above (Figure 10h), compounds C45897625 and C81903127 exhibited deficiencies in critical hydrogen bonding interactions, while compounds 1_48 and 1_99 lacked key hydrophobic interactions. Additionally, compound C81903127 did not meet the required docking score. Consequently, a total of 11 compounds were screened, with an accuracy of 100% and a true positive rate of 84.6% (Figure 10i).

**Figure 11:**
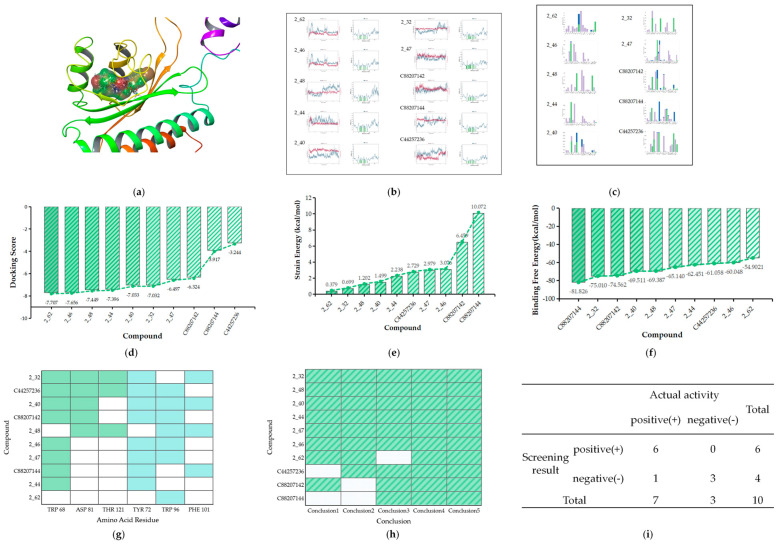
(**a**) The 3D docking diagram of test set of Class 2 (**b**) RMSD and RMSF plots of test set (**c**) Interaction histograms of test set (**d**) The ranking of compounds in Class 2 based on docking scores (**e**) Strain energy of test set of Class 2 (**f**) Binding free energy of test set of Class 2 (**g**) The matches of interactions with the standard of Class 2 (the blocks with green represent hydrogen bonding, while blue signify hydrophobic interactions) (**h**) The summary of screening results for test set of Class 2 (the blocks with green represents compliance with standards, while the absence of a color indicates non-compliance) (**i**) The screening performance of the model was validated by the test set of Class 2.

The same workflow of virtual screening is employed to validate the test set in Class 2 at the level of interaction and affinity with the receptors (Figure 11a–c). The docking scores shown in Figure 11d indicate that compounds C88207144 and C44257236 do not meet the criterion of having a docking score less than −6 and are thus excluded. Moreover, as shown in Figure 11e, compounds C88207144 and C88207142 have strain energies exceeding 4 kcal/mol, thus failing to meet the requirements for conformational changes.

The molecular dynamics results showed that all compounds in the test set, except compound 2_62, exhibit relatively stable interactions with the receptor and are compliant with the conditions outlined in Section 2.2.2 (Figure 11g). All compounds in the test set meet the criteria for binding free energy, as shown in Figure 11f.

In summary, compounds C88207144 and C44257236 did not satisfy the required standards for docking scores. In addition, compounds C88207144 and C88207142 fall short of the threshold for strain energy. Moreover, critical hydrogen bonds are absent in 2_62 (Figure 11h). Consequently, six compounds were selected through the screening process of Class 2, with an accuracy of 100% and a true positive rate of 85.7% (Figure 11i).

Thus, the systematic hierarchical virtual screening model for RhlR inhibitors developed in this study, integrating pharmacophores, molecular docking, molecular dynamics simulation, and the calculation of binding free energy and strain energy, effectively distinguishes active RhlR inhibitors.

### 2.5. Validation and Comparison of Test Sets against Existing Screening Models

**Figure 12:**
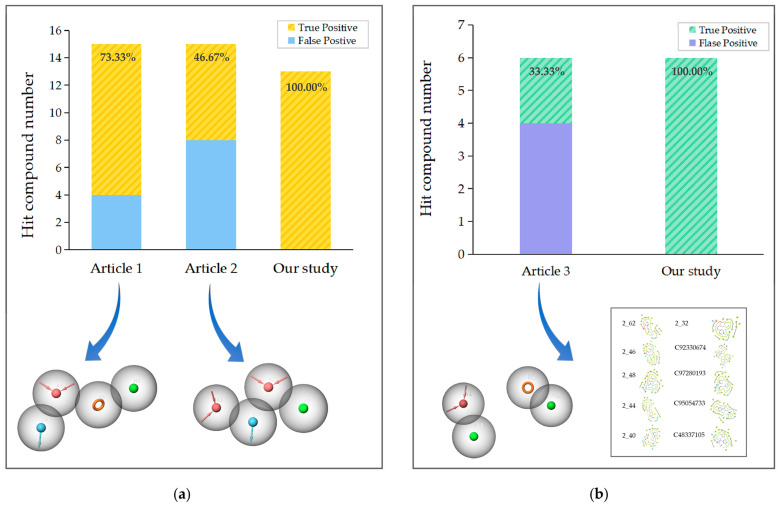
(**a**) The comparison of different screening criteria for Class 1, with yellow bars presenting the number of true positive compounds, while blue representing the number of false positives. (Article 1: [25]; Article 2: [23].) (**b**) The comparison of different screening criteria for Class 2, with green bars presenting the number of true positive compounds, while purple representing the number of false positives. (Article 3: [27]).

To further validate the advancement of our model, a comparative analysis was performed between the existing screening criteria for two classes of compounds and the virtual screening model developed in this study. The test set utilized in this experiment was subjected to the aforementioned pharmacophores belonging to Class 1. Within the top-ranked compounds screened by the ADRH pharmacophore, 11 compounds were actually active compounds, achieving 73.33% accuracy. As for the other pharmacophore, AADH, the screening results indicate an accuracy of 46.67% (Figure 12a).

Similarly, we applied the test set to the pharmacophore model AHHR of Class 2 and selected the top-ranked compounds. Next, we docked these compounds with the homologous RhlR receptor used in the original lecture. The docking results showed that compounds 2_46, 2_32, C92330674, C97280193, C95054733, and C48337105 met the key interactions described in the lecture and are thus considered active (Figure S7). Eventually, the accuracy and true positive rate will be 33.33% (Figure 12b).

The comparative results of the test sets suggested that both types of virtual screening models developed in this study demonstrated a higher accuracy and true positive rate.

Consequently, the systematic hierarchical virtual screening model, integrating pharmacophore, docking, molecular dynamics, calculations of binding free energy, and strain energy, can identify more accurately and thus offer insights into the development of subsequent RhlR inhibitors.

## 3. Discussion

In this study, a systematic hierarchical virtual screening model for RhlR inhibitors was developed by integrating virtual screening techniques across various levels, including ligand structure, ligand–receptor interactions, and binding affinity.

To investigate the characteristics of existing RhlR inhibitors, we employed principal component analysis (PCA) to categorize the inhibitors into two classes. Subsequently, SAR analysis revealed that the two classes of compounds are, respectively, derived from two parent nuclei: BHL and gingerol. The primary feature of Class 1 is the presence of an amide bond in the central region, while Class 2 features a central region comprising either a ketone carbonyl or a hydroxyl group. The results of SAR validated the rationality of the classification by PCA. Following that, pharmacophore models were developed based on the training sets to analyze the characteristic structure of ligands. Pharmacophores AAADR_1 and AHHR_2 were, respectively, established for each class. The differences between the two pharmacophore models demonstrated structural distinctions between the two classes. The first class exhibits a higher propensity for hydrogen bond acceptors or donors, while the second class contains more hydrophobic elements. It can be observed that both classes essentially feature benzene rings. The amide bond, a characteristic structural feature of Class 1 described in the SAR, corresponds to the specific element hydrogen bond donor D of the pharmacophore. Additionally, in the head region, the lactone ring present in Class 1 corresponds to two hydrogen bond acceptor elements (A), while the carbon chain in Class 2 corresponds to the hydrophobic element (H). Moving forward, molecular docking, molecular dynamics simulations, and the calculation of binding free energy and strain energy were performed to explore the receptor–ligand interaction and receptor affinity. Ultimately, as listed in Section 2.2.2, distinct screening criteria were established for the two classes of inhibitors. By comparing the two screening models, we found that for Class 1, unique key interactions appear to be an important aspect of distinguishing active compounds from inactive compounds. Whereas for Class 2, the binding affinity with the receptor is the discriminant. This can be explained by the fact that the pharmacophore model of Class 1 has more hydrogen bond forming elements. Interestingly, there are differences in amino acids forming hydrogen bonds between the two classes. The unique hydrogen bonding in Class 1 involves TYR 64 and SER 135, while compounds in Class 2 interact via unique hydrogen bonds with THR_121. It is imperative to note that a large number of active compounds in both classes form hydrogen bond interactions with TRP_68 and ASP_81. Therefore, these two amino acid residues may be crucial for the binding of compounds to the RhlR receptor. In addition, both classes also engage in hydrophobic interactions with TYR_72, TRP_96, and PHE_101, which often occur at the aromatic ring element (R) of pharmacophores. This suggests that subsequent inhibitor development can focus on the hydrogen bond interactions of TRP_68 and ASP_81, as well as the hydrophobic interactions involving TYR_72, TRP_96, and PHE_101. Both classes of compounds primarily form crucial hydrogen bonds through central functional groups. Due to structural differences, interactions in Class 1 typically occur at the amide bond, while in Class 2, they often involve the central hydroxyl group.

To further assess our model, we compared it with existing studies. Focusing on Class 1, there have been studies elucidating the SAR but lacking descriptions of critical interactions. Boursier et al. suggested that inhibitory activity is influenced by a tetrahydrofurfuryl amine head group, a sulphonamide moiety, and a tail containing either a quaternary or sp2 hybridized α-carbon [23]. On the other hand, Eibergen et al. found that compounds with halogen-substituted aromatic groups were the most effective inhibitors [25]. Based on the aforementioned SAR descriptions, we constructed the pharmacophore models AADH and ADRH accordingly. The screening results indicated the current models have an accuracy of 46% and 73%, whereas our research achieved 100%. Our screening model exhibited higher accuracy, potentially attributed to the exploration of receptor–ligand interactions, as well as the inclusion of a larger set of compounds. Additionally, comparative results among different pharmacophores indicated that incorporating the aromatic ring element could enhance screening accuracy, implying that retaining the benzene ring might be more beneficial for modifying active compounds.

Research has currently discussed the structural characteristics and key interactions of Class 2. SangJin Nam et al. reported that active inhibitors often contain phenyl rings with polar substituents, carbonyl or hydroxyl groups at the γ-position of the phenyl ring, and terminal alkyl chains [27]. They also pointed out that active compounds tend to interact with the amino acid residues TYR 72 and TRP 68. A tiered screening model has been established based on the conclusions above, with a screening accuracy of 33.3%. and a true positivity rate of 33.3%. This is lower than that of our model, which achieved 100% and 85.7%. The reason lies in the exploration method of key interactions. The critical interactions discussed in the reference articles were based on an individual compound within a static environment, whereas our model simulated the dynamic interactions between ligands and receptors, thus allowing for higher accuracy. Moreover, our model also characterizes the affinity between ligands and receptors.

In contrast to existing screening criteria, this study analyzes the differences between active and inactive compounds based on ligand structure, ligand–receptor interactions, and receptor affinity (binding free energy), providing comprehensive information for the screening of active RhlR inhibitors. The comparative results showed that both of our models demonstrated higher screening performance, suggesting the need to consider multiple dimensions in the establishment of virtual screening models.

In this study, we developed a systematic, hierarchical virtual screening model for RhlR inhibitors by integrating ligand structure, ligand–receptor interactions, and binding affinity. This model has significant practical implications for predicting active compounds and can contribute to the future development of novel RhlR inhibitors. In future studies, we plan to use this model to screen compounds and guide their modification. Enzyme and biofilm experiments will also be conducted to validate the model and determine the activity of compounds. Based on this, the model can expand the inhibitor database and facilitate optimizations, including ligand-based and ligand–receptor complex aspects. Ultimately, our goal is to further refine the virtual screening model and establish a closed-loop system encompassing modeling, screening, validation, and optimization.

## 4. Materials and Methods

### 4.1. Establishment and Preparation of Database

We conducted a search using the keywords “RhlR inhibitor” and “RhlR antagonist” in two literature databases: PubMed and Web of Science. The inclusion and exclusion criteria for this study were defined as follows:

1. The article must focus on RhlR inhibitors.
2. The study must experimentally evaluate the efficacy of compounds in inhibiting RhlR, along with corresponding activity data.
3. The article should include a discussion on the structure–activity relationship of RhlR inhibitors.

We initially retrieved 107 articles, from which 14 were selected based on abstract screening. However, after thorough review, only 5 articles met the inclusion criteria (Table S1). All other references, apart from the 5 included, are presented in Supplementary Materials [35,41,42,43,44,45,46,47,48,49,50,51,52,53,54,55,56,57,58,59,60,61,62,63,64,65,66,67,68,69,70,71,72,73,74,75,76,77,78,79,80,81,82,83,84,85,86,87,88,89,90,91,92,93,94,95,96,97,98,99,100,101,102,103,104,105,106,107,108,109,110,111,112,113,114,115,116,117,118,119,120,121,122,123,124,125,126,127,128,129,130,131,132,133,134,135,136,137,138,139,140,141]. We assembled a database comprising 162 compounds sourced from the literature that met the criteria above [23,24,25,26,27]. The inhibition values for these compounds were integrated into the database, and subsequently, a database of RhlR inhibitors was set up. This was achieved by converting all activity data to inhibition rates at a concentration of 100 μM relative to the standard control, utilizing compound D8 as a reference, which was assayed under both conditions [23,25]. All compounds underwent preparation using the LigPrep module, maintaining neutral ligand ionization states and minimizing their energies under the OPLS4 force field. This ensured the generation of corresponding low-energy 3D structures for all compounds [142].

### 4.2. PCA and Systematic Review

Molecular descriptors were initially generated for each compound using the Molecular Descriptors module, and were then ranked based on ANOVA. The top 20 descriptors were selected as the original variables for PCA. The PCA was conducted using the R programming language. The baseline table for article assessment was completed by extracting the following key information.

1. Strain
2. Activity evaluation index
3. Method used for evaluating the structure–effect relationship
4. Description of the structure–effect relationship

### 4.3. Software Information

Maestro 13.5 software was utilized for modeling and validation screening. The “Develop Pharmacophore Hypothesis” panel was employed for pharmacophore modeling, while the “Ligand and Database Screening” module was utilized for screening and validating the pharmacophore. The “Ligand Docking” panel was utilized to establish and validate criteria for molecular docking, while the “System Builder”, “Minimization”, and “Molecular Dynamics” tools were employed to establish and validate criteria for molecular dynamics. The MM-GBSA method was employed for calculating binding free energy. The “Strain Energy Calculation and Rescoring” module was used to compute strain energy. All the compound structures were constructed using ChemDraw 19.0.

### 4.4. Construction of Pharmacophore Models

Ligand-based pharmacophore modeling was conducted using the Develop Pharmacophore Hypothesis module. The model encompasses six pharmacophore characteristics: hydrogen bond acceptor (A), hydrogen bond donor (D), aromatic ring (R), hydrophobicity (H), positively charged group, and negatively charged group. Here, the aromatic ring is considered the hydrophobic ring, while the negatively charged group serves as the acceptor. Parametric metrics, including the receiver operating characteristic (ROC) curve, enrichment factor at 1% (EF1%), area under the curve (AUC), BEDROC160.9, and average outranking decoys, were assessed to identify the optimal pharmacophore model. The ROC curve represents the likelihood of ranking compounds with known activity higher than decoys when selected randomly [143], with values approaching one indicating a more reliable pharmacophore model. EF1% evaluates the enrichment of active compounds within the top 1% of the screening library [144].

### 4.5. Protein Preparation and Molecular Docking

The RhlR protein (PDB ID: 8B4A) was obtained from https://www.rcsb.org (accessed on 15 July 2023), and only the D chain was retained as the receptor. The receptor protein underwent processing using the Protein Preparation tool of Maestro 13.5, with the constraint of retaining amino acids within a 5 Å radius of the docking center as rotatable.

Compounds were subjected to molecular docking to elucidate the binding interactions between ligands and amino acid residues. Molecular docking was conducted using the Ligand Docking module, with compounds imported into the docking box previously generated as receptors. During the docking process, the XP mode was selected to enhance the precision of docking bonding patterns, the flexible mode was enabled for flexible docking, and the scaling factor was set to 0.80. Ten conformations were generated for each compound. In alignment with the crystal compound’s conformation, the conformation with the highest docking score was selected as the optimal conformation.

### 4.6. Molecular Dynamics Simulations, Calculation of Binding Free Energy and Strain Energy

Given the dynamic variability in interactions between compounds and receptors, molecular dynamics simulations are utilized to elucidate the interactions between ligands and proteins in the motile state. The receptor protein environment is established using the System Builder module, followed by energy minimization using the Minimization module. Subsequently, the Molecular Dynamics module is employed to simulate the dynamics of the compounds throughout the 100 ns. The stability of proteins and ligands is assessed through RMSD (root mean square deviation) and RMSF (root mean square fluctuation): RMSD illustrates the displacement of frame at various time points relative to the initial moment, with fluctuations of less than 3 Å indicating relative stability in conformation for both receptors and ligands; RMSF delineates the local changes along the protein backbone; and the green-colored vertical bars mark the interactions between residues and ligands. The protein-ligand contact diagram is employed to visually assess the strength of various interactions between amino acid residues, with interactions exceeding 0.4 in value considered relatively strong. Additionally, binding free energy and strain energy are calculated to investigate the binding affinity. The time for binding free energy ranges from 80 to 100 ns. In the calculation of strain energy, the chosen solvent is 4RDDD (4r distance-dependent dielectric), with the energy offset for strain correction set to the default value.

### 4.7. Virtual Screening Validation of the Test Set

In the validation of our model, the pharmacophore underwent validation using the Ligand and Database Screening module. The test set was introduced into the established pharmacophore, and half of the active test set was selected based on PhaseScore rankings. Docking of the test set was performed using the same docking box and parameters as utilized in the training set. Molecular dynamics simulations were conducted under identical conditions.

In comparison with existing models, pharmacophore models were established for articles solely describing SAR at the ligand level. The models were fed with the same test set. Similarly, top-ranked compounds were selected to investigate the accuracy of activity inhibitors. Accuracy was then employed as the metric to evaluate the model. For articles also describing receptor–ligand interactions, in addition to pharmacophore screening, further hierarchical selection was conducted based on docking descriptions. Compounds satisfying the critical interactions were considered active. Accuracy and the true positive rate were calculated from the screening results and used as comparative benchmarks.

## Data Availability

Data presented in this study are available on request from the corresponding author.

## Supplementary Materials

The following supporting information can be downloaded at: https://www.mdpi.com/article/10.3390/ijms25148000/s1.

## Abbreviations

Explanation of abbreviations in the text.

**Abbreviations**	**Full Names**
PCA	Principal component analysis
SAR	Structure-activity relationship
QS	Quorum sensing
AHL	Acyl-homoserine lactone
QSIs	Quorum sensing inhibitors
BHL	N-butyryl-L-homoserine lactone
PCs	Principal components
A	Hydrogen bond acceptor
D	Hydrogen bond donor
R	Aromatic ring
H	Hydrophobic group
ROC	Receiver operating characteristic
EF1%	Enrichment Factor at 1%
AUC	Area Under the Curve
AUAC	Area Under the Absolute Calibration Curve
RMSD	Root mean square deviation
RMSF	Root mean square fluctuation
4RDDD	4r distance-dependent dielectric
