# Supplementary material for: A Systematic Hierarchical Virtual Screening Model for RhlR Inhibitors Based on PCA, Pharmacophore, Docking, and Molecular Dynamics
Source: Int J Mol Sci. 2024 Jul 22;25(14):8000. doi: 10.3390/ijms25148000 (PMC11276863; doi:10.3390/ijms25148000)
Supplement: Supplementary file 1 [file ijms-25-08000-s001.zip › ijms-3086783-supplementary.pdf]

Table S1. Compliance of literature and Inclusion Exclusion Criteria

Reference	Criteria 1[1]	Criteria 2[2]	Criteria 3[3]	Include or exclude
[41]	×*	√	×	exclude
[40]	√*	×	√	exclude
[25]	√	√	√	include
[27]	√	√	√	include
[42]	×	√	×	exclude
[23]	√	√	√	include
[24]	√	√	√	include
[26]	√	√	√	include
[43]	×	×	×	exclude
[44]	×	√	×	exclude
[45]	×	√	×	exclude
[46]	×	×	√	exclude
[47]	×	×	×	exclude
[48]	×	×	×	exclude

[1] The article must focus on RhlR inhibitor.
[2] The study must experimentally evaluate the efficacy of compounds in inhibiting RhlR, along with corresponding activity data.
[3] The article should include a discussion on the structure-activity relationship of RhlR inhibitors.

* "×" indicate non-compliance with the criteria, while "√" indicate compliance with the criteria.

Table S2. The top 20 molecular descriptors ranked by Analysis of Variance (ANOVA)

NO.	Molecular Descriptors
1	i_desc_Atoms_in_Ring_System
2	i_desc_Bonds_in_Ring_System
3	i_desc_Cyclomatic_number
4	i_desc_Number_of_ring_systems
5	i_desc_Ring_Count_5
6	i_desc_Ring_perimeter
7	i_desc_Total_ring_size
8	r_desc_ALOGP3
9	r_desc_Average_connectivity_index_chi-1
10	r_desc_Average_connectivity_index_chi-3
11	r_desc_Balaban-type_index_from_Z_weighted_distance_matrix_-_Barysz_matrix
12	r_desc_Balaban-type_index_from_electronegativity_weighted_distance_matrix
13	r_desc_Balaban-type_index_from_mass_weighted_distance_matrix
14	r_desc_Balaban-type_index_from_polarizability_weighted_distance_matrix
15	r_desc_Balaban-type_index_from_van_der_waals_weighted_distance_matrix
16	r_desc_Balaban_distance_connectivity_index
17	r_desc_Molecule_cyclized_degree
18	r_desc_PEOE13
19	r_desc_PEOE2
20	r_desc_Spanning_tree_number

Table S3 . Database of RhlR inhibitor

Name	Structure	From	Name	Structure	From	Name	Structure	From	Name	Structure	From	Name	Structure	From
1_8	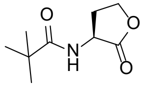	1	1_66	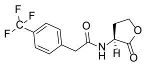	3	1_106	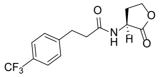	3	2_6	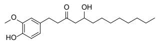	5	2_39	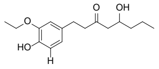	5
1_9	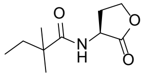	1	1_67	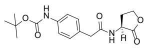	3	1_107	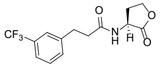	3	2_7	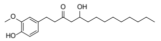	5	2_40	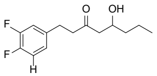	5
1_10	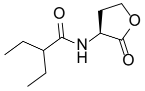	1	1_68	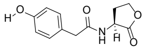	3	1_110	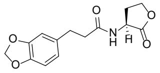	3	2_8	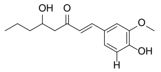	5	2_41	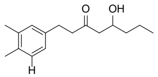	5
1_12	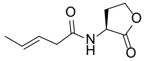	1	1_69	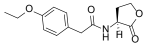	3	1_111	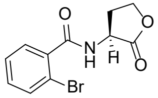	3	2_9	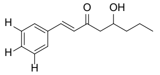	5	2_42	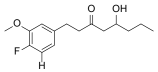	5
1_18	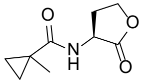	1	1_70	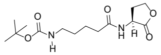	3	1_112	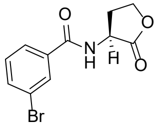	3	2_10	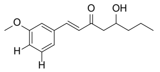	5	2_43	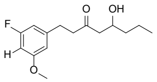	5
1_19	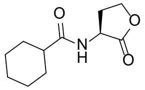	1	1_71	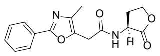	3	1_113	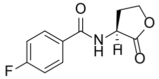	3	2_11	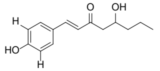	5	2_44	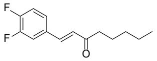	5
1_20	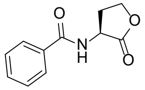	1	1_72	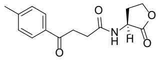	3	1_114	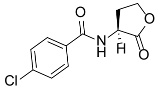	3	2_12	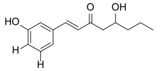	5	2_45	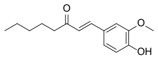	5

1_21	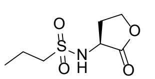	1	1_73	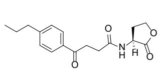	3	1_115	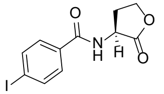	3	2_13	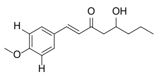	5	2_46	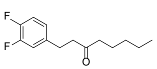	5
1_22	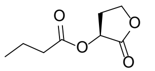	1	1_74	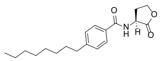	3	1_116	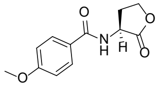	3	2_14	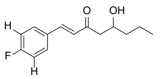	5	2_47	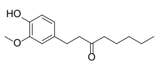	5
1_23	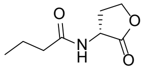	1	1_75	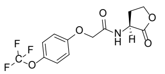	3	1_117	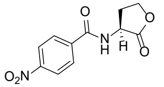	3	2_15	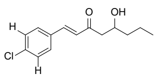	5	2_48	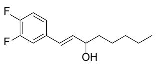	5
1_25	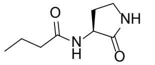	1	1_76	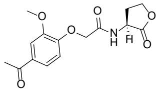	3	1_118	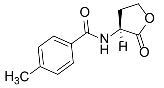	3	2_16	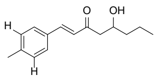	5	2_49	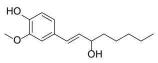	5
1_27	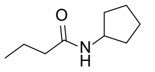	1	1_77	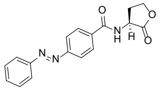	3	1_120	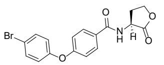	3	2_17	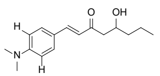	5	2_50	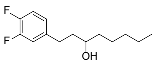	5
1_29	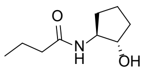	1	1_81	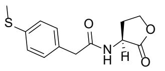	3	1_121	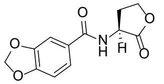	3	2_18	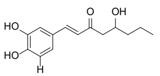	5	2_51	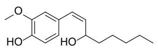	5
1_31	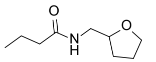	1	1_83	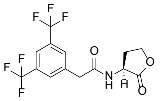	3	1_122	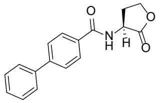	3	2_19	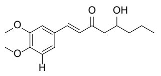	5	2_52	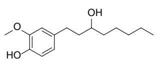	5
1_36	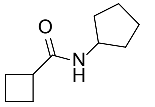	2	1_84	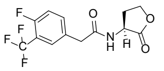	3	1_123	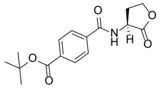	3	2_20	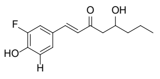	5	2_53	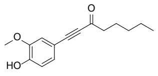	5

1_37	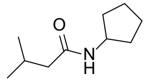	2	1_85	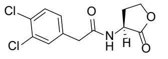	3	1_124	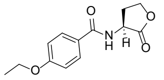	3	2_21	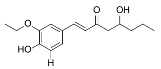	5	2_54	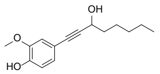	5
1_38	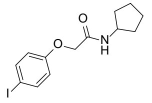	2	1_86	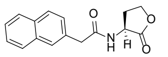	3	1_125	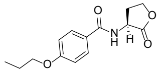	3	2_22	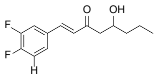	5	2_55	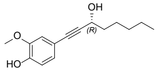	5
1_39	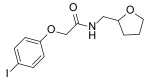	2	1_88	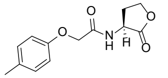	3	1_126	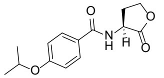	3	2_23	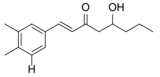	5	2_56	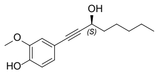	5
1_40	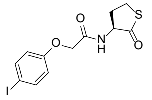	2	1_90	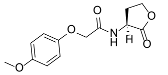	3	1_127	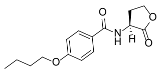	3	2_24	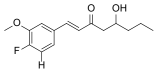	5	2_57	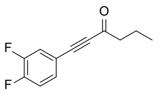	5
1_41	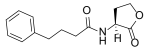	3	1_91	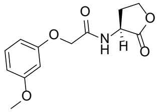	3	1_128	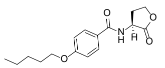	3	2_25	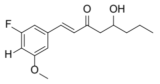	5	2_58	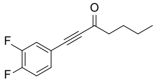	5
1_42	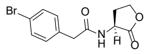	3	1_93	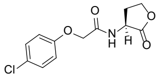	3	1_130	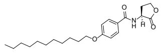	3	2_26	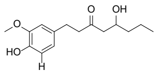	5	2_59	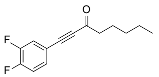	5
1_43	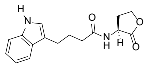	3	1_94	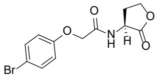	3	1_131	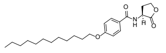	3	2_27	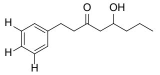	5	2_60	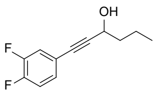	5
1_44	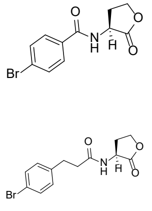	3	1_95	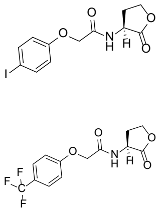	3	1_132	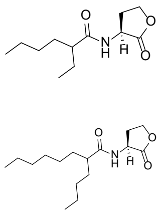	3	2_28	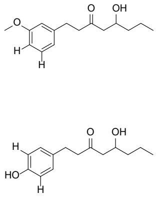	5	2_61	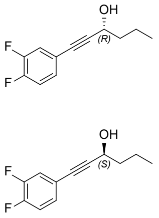	5
1_45		3	1_96		3	1_133		3	2_29		5	2_62		5

1_46	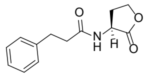	3	1_97	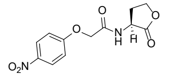	3	1_134	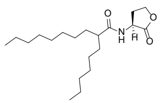	3	2_30	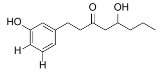	5	2_63	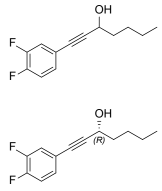	5
1_47	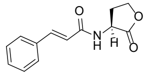	3	1_98	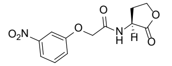	3	1_135	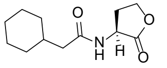	3	2_31	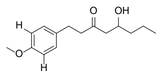	5	2_64		5
1_48	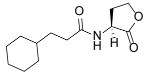	3	1_99	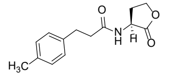	3	1_137	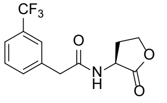	4	2_32	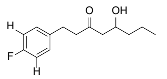	5	2_65	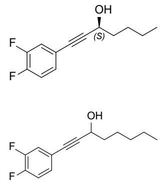	5
1_57	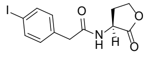	3	1_100	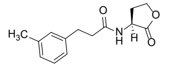	3	1_138	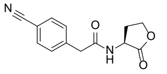	4	2_33	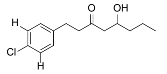	5	2_66		5
1_60	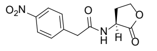	3	1_101	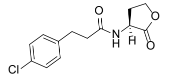	3	2_1	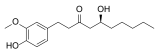	5	2_34	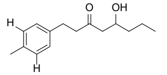	5	2_67	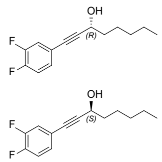	5
1_62	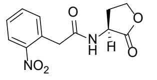	3	1_102	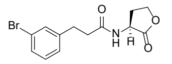	3	2_2	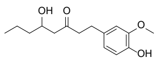	5	2_35	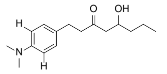	5	2_68		5
1_63	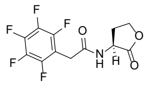	3	1_103	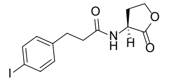	3	2_3	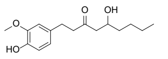	5	2_36	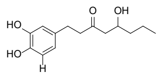	5			
1_64	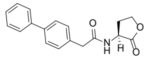	3	1_104	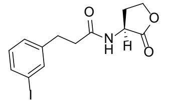	3	2_4	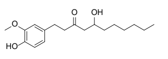	5	2_37	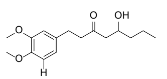	5			
1_65	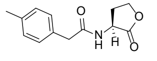	3	1_105	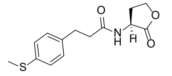	3	2_5	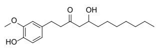	5	2_38	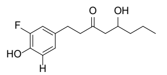	5			

Table S4. Baseline analysis table of included literature

NO.	Reference	Activity evaluation indicators	Evaluation method	Structure-Activity Relationship
1	[23]	Inhibition of compounds screened at 10 μM in the presence of 10 μM BHL	Chemistry synthesis; Comparative analysis	①Compounds with quaternary ammonium or sp2 hybridized carbons have a volume of space close to the HL, yielding weak antagonistic activity. ②Sulfonamides act antagonistically by increasing the volume of space adjacent to the amide NH. ③The localization of the derived tetrahydrofurfuryl head strengthens the inhibitory interaction. ④Removing or altering many of the aforementioned critical components of BHL for RhlR activation results in RhlR antagonists.
2	[24]	Inhibition of compounds screened at 10 μM in the presence of 10 μM BHL;IC50	Chemistry synthesis;Comparative analysis	①Homoserine lactone headgroups can enhance the efficacy of inhibiting RhlR. ②Homocysteine thiolactone derivative(aryl thiolactone 42) has strong potency and efficacy comparable to its parent aryl lactone E22, and cyclopentyl derivative 38 prove to be the next most active.
3	[25]	Inhibition of compounds evaluated at 100 μM in the presence of 10 μM BHL;<br>IC50	E. coli reporter gene assays，P. aeruginosa reporter assays	①Almost all RhlR antagonists contain an aromatic acyl group, with the exception of cyclohexyl, these RhlR antagonists belonged to the PHL, PPHL, or phenoxyacetyl homoserine lactone (POHL) classes. ②The most defining characteristic is substitution at the para position of their aromatic groups, especially in the POHL and PPHL structural classes, and these substituents are relatively diverse in size and electronic nature (ranging from 2,3,4,5,6-pentafluoro to 2-napthyl). ③L-lactone stereochemistry is not a requirement for RhlR antagonism by AHLs. ④The PHLs and POHLs with halogen-substituted aromatic groups tend to display the strongest RhlR antagonism.
4	[26]	GFP expression by PAO-JP2 (prhlI-LVAgfp) in the presence of 1 μM AI1 and 10 μM AI2	Chemistry synthesis	The chain length appears to be the determining factor regulating RhlR activation

5	[27]	Inhibition of compounds evaluated at 100 μM in the presence of 10 μM BHL;<br>IC50	Chemistry synthesis;<br>Silico molecular docking;<br>Comparative analysis	① Gingerols with the shorter alkyl chain have a higher affinity for RhlR;<br>② A polar functional group in the phenyl ring is required for binding to RhlR;<br>③It is necessary that there are small and polar groups (i.e., −F and −OH) at 4′-position.<br>④The larger the substituent group at 3′-position is, the weaker the antagonism is.<br>⑤The F-substitution in the phenyl ring is preferred for the structural modification of the phenyl ring in monosubstituted and disubstituted analogues.<br>⑥The compounds with a double bond displayed stronger RhlR antagonism than those with a single bond, irrespective of the substituents in the phenyl ring.<br>⑦The carbonyl group or the hydroxyl group at the γ-position from the phenyl group is important for binding to RhlR.<br>⑧The compounds with the shorter alkyl chain length were stronger than the corresponding ones with the longer alkyl chain lengths.

Table S5. (a) Training set of Class 1 (b)Training set of Class 2

Active		Inactive	
NO.	Name	NO.	Name
1	1_36	11	1_8
2	1_40	12	1_23
3	1_57	13	1_25
4	1_65	14	1_31
5	1_66	15	1_83
6	1_81	16	1_102
7	1_86	17	1_122
8	1_88	18	1_130
9	1_95	19	1_131
10	1_135	20	1_133

Active		Inactive	
NO.	Name	NO.	Name
1	2_14	11	2_4
2	2_22	12	2_5
3	2_53	13	2_9
4	2_54	14	2_10
5	2_55	15	2_21
6	2_57	16	2_27
7	2_58	17	2_28
8	2_59	18	2_37
9	2_60	19	2_39
10	2_61	20	2_43

Table S6. (a) Test set of Class 1 (b) Test set of Class 2

Active					
**NO.**	**Compound**	**NO.**	**Compound**	**NO.**	**Compound**
1	1_12	11	1_62	21	1_96
2	1_21	12	1_63	22	1_97
3	1_27	13	1_68	23	1_98
4	1_37	14	1_69	24	1_99
5	1_42	15	1_72	25	1_103
6	1_43	16	1_75	26	1_105
7	1_44	17	1_85	27	1_106
8	1_47	18	1_90	28	1_114
9	1_48	19	1_93	29	1_115
10	1_60	20	1_94	30	1_138
Decoy					

Active			
**NO.**	**Compound**	**NO.**	**Compound**
1	2_2	11	2_40
2	2_6	12	2_44
3	2_7	13	2_45
4	2_11	14	2_46
5	2_13	15	2_47
6	2_17	16	2_48
7	2_18	17	2_49
8	2_24	18	2_52
9	2_32	19	2_56
10	2_36	20	2_62
Decoy			

Table S7. (a) The ranking of pharmacophores of Class 1 (b) The ranking of pharmacophores of Class 2

(a)

Hypothesis	Phase Hypo Score	EF1%	BEDROC 160.9	ROC	AUAC	Ave Outranking Decoys	Total Actives	Ranked Actives	Matches	Excluded Volumes
AAADR_1	1.14	90.9	0.96	0.9	0.93	0	10	9	5 of 5	No
AAADR_2	1.14	90.9	0.96	0.9	0.93	0	10	9	5 of 5	No
AAADR_3	1.13	90.9	0.96	0.9	0.93	0	10	9	5 of 5	No
AAADH_1	1.13	90.9	0.96	0.9	0.93	0	10	9	5 of 5	No
AAADR_4	1.13	90.9	0.96	0.9	0.93	0	10	9	5 of 5	No
AAADR_5	1.13	90.9	0.96	0.9	0.93	0	10	9	5 of 5	No
AAADR_6	1.13	90.9	0.96	0.9	0.93	0	10	9	5 of 5	No
AAADR_7	1.13	90.9	0.96	0.9	0.93	0	10	9	5 of 5	No
AAADR_8	1.13	90.9	0.96	0.9	0.93	0	10	9	5 of 5	No
AAADR_9	1.13	90.9	0.96	0.9	0.93	0	10	9	5 of 5	No
AAAR_1	1.13	90.9	0.96	0.9	0.92	0	10	9	4 of 4	No
AAAH_1	1.13	90.9	0.96	0.9	0.92	0	10	9	4 of 4	No
AADR_2	1.13	90.9	0.96	0.9	0.92	0	10	9	4 of 4	No
AADR_3	1.13	90.9	0.96	0.9	0.92	0	10	9	4 of 4	No
AADR_4	1.13	90.9	0.96	0.9	0.92	0	10	9	4 of 4	No
AADR_5	1.13	90.9	0.96	0.9	0.92	0	10	9	4 of 4	No
AADR_1	1.13	90.9	0.95	0.9	0.92	0.11	10	9	4 of 4	No
AAAR_2	1.13	90.9	0.95	0.9	0.92	0.11	10	9	4 of 4	No
AAAR_3	1.12	90.9	0.93	0.9	0.92	0.33	10	9	4 of 4	No
AAAR_4	1.11	80.8	0.92	0.9	0.92	0.89	10	9	4 of 4	No

(b)

Hypothesis	Phase Hypo Score	EF1%	BEDROC 160.9	ROC	AUAC	Ave Outranking Decoys	Total Actives	Ranked Actives	Matches	Excluded Volumes
AHHR_2	1.08	70.7	0.86	0.98	0.98	19.9	10	10	4 of 4	No
AHHHR_1	0.89	60.6	0.77	0.6	0.74	0	10	6	5 of 5	No
AHHHR_2	0.89	60.6	0.77	0.6	0.74	0	10	6	5 of 5	No
AHHHR_4	0.89	60.6	0.77	0.6	0.74	0	10	6	5 of 5	No
AHHHR_5	0.89	60.6	0.77	0.6	0.74	0	10	6	5 of 5	No
AHHHR_6	0.88	60.6	0.77	0.6	0.74	0	10	6	5 of 5	No
AHHHR_7	0.88	60.6	0.77	0.6	0.73	0	10	6	5 of 5	No
AHHHR_8	0.88	60.6	0.77	0.6	0.75	0	10	6	5 of 5	No
AHHHR_9	0.88	60.6	0.77	0.6	0.74	0	10	6	5 of 5	No
AHHHR_10	0.88	60.6	0.77	0.6	0.74	0	10	6	5 of 5	No
AHHHR_3	0.88	60.6	0.74	0.6	0.74	0.5	10	6	5 of 5	No
AHHR_1	0.87	60.6	0.77	0.6	0.71	0	10	6	4 of 4	No
HHHR_1	0.86	60.6	0.77	0.6	0.71	0	10	6	4 of 4	No
HHHR_2	0.86	60.6	0.77	0.6	0.71	0	10	6	4 of 4	No
HHHR_3	0.86	60.6	0.77	0.6	0.72	0	10	6	4 of 4	No
HHHR_4	0.86	60.6	0.77	0.6	0.7	0	10	6	4 of 4	No
HHHR_5	0.86	60.6	0.77	0.6	0.7	0	10	6	4 of 4	No
HHHR_6	0.86	60.6	0.77	0.6	0.7	0	10	6	4 of 4	No
HHHR_8	0.86	60.6	0.77	0.6	0.71	0	10	6	4 of 4	No
HHHR_7	0.86	60.6	0.76	0.6	0.7	0.17	10	6	4 of 4	No

(EF1%: Enrichment Factor at 1%, ROC: Receiver operating characteristic, AUAC: Area Under the Absolute Calibration Curve)

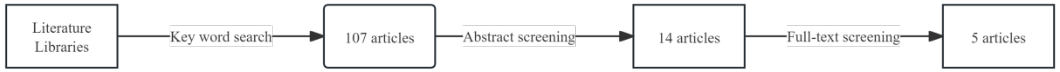
Literature Libraries
Key word search
107 articles
Abstract screening
14 articles
Full-text screening
5 articles
